# E-Cigarette Markets and Policy Responses in Southeast Asia: A Scoping Review

**DOI:** 10.34172/ijhpm.2021.25

**Published:** 2021-04-13

**Authors:** Yvette van der Eijk, Grace Tan Ping Ping, Suan Ee Ong, Grace Tan Li Xin, David Li, Dijin Zhang, Loo Min Shuen, Chia Kee Seng

**Affiliations:** ^1^Saw Swee Hock School of Public Health, National University of Singapore, Singapore, Singapore.; ^2^Research for Impact, Singapore, Singapore.; ^3^Department of Political Science, Faculty of Arts and Social Sciences, National University of Singapore, Singapore, Singapore.; ^4^Department of Civil and Environmental Engineering, Faculty of Engineering, University of Alberta, Edmonton, AB, Canada.; ^5^Department of Biological Sciences, Faculty of Science, National University of Singapore, Singapore, Singapore.; ^6^Department of Psychology, Faculty of Arts and Social Sciences, National University of Singapore, Singapore, Singapore.

**Keywords:** E-Cigarettes, Industry, Health Policy, Nicotine, Tobacco, Vaping

## Abstract

**Background:** The global e-cigarette market has proliferated and is increasingly dominated by transnational tobacco companies. While Southeast Asian countries have received relatively little attention in e-cigarette research, the region represents an area of potentially untapped growth for the industry. We review the e-cigarette situation in Southeast Asia in terms of the e-cigarette markets, advertising and promotion of e-cigarettes, reported health impacts of e-cigarette use, and policy responses in the region.

**Methods:** We examined e-cigarette market data from the Euromonitor Global Market Information Database (GMID) Passport database, searched in the academic literature, grey literature and news archives for any reports or studies of e-cigarette related diseases or injuries, e-cigarette marketing, and e-cigarette policy responses in Southeast Asian countries, and browsed the websites of online e-cigarette retailers catering to the region’s active e-cigarette markets.

**Results:** In 2019, e-cigarettes were sold in six Southeast Asian markets with a total market value of $595 million, projected to grow to $766 million by 2023. E-commerce is a significant and growing sales channel in the region, with most of the popular or featured brands in online shops originating from China. Southeast Asian youth are targeted with a wide variety of flavours, trendy designs and point of sale promotions, and several e-cigarette related injuries and diseases have been reported in the region. Policy responses vary considerably between countries, ranging from strict bans to no or partial regulations.

**Conclusion:** Although Southeast Asia’s e-cigarette market is relatively nascent, this is likely to change if transnationals invest more heavily in the region. Populous countries with weak e-cigarette regulations, notably Indonesia, Malaysia, Vietnam and the Philippines, are desirable targets for the transnationals. Regulatory action is needed to prevent e-cigarette use from becoming entrenched into these societies, especially among young people.

## Introduction

 E-cigarettes are devices that heat a solution, ‘e-liquid,’ to create an aerosol which is inhaled (‘vaped’). Although the earliest e-cigarette devices resembled cigarettes, many of the newer varieties take on a wide range of forms, often resembling pens, tech gadgets, and other everyday items. The e-liquids, which usually (but not always) contain nicotine, frequently contain flavours and additives, usually dissolved into a propylene glycol or glycerine solution. E-cigarettes are therefore not a homogenous group of products but a broad category of devices, e-liquids, and product components that, when assembled, are intended to be used in a similar manner as tobacco products.^1^ The global e-cigarette market has proliferated from around $50 million (USD) in 2005 to over $20 billion in 2019, and is expected to grow to $34 billion by 2024.^2^

 E-cigarettes remain controversial as the evidence base remains inconclusive on their long-term safety and effectiveness as a smoking cessation aid. Among recent studies published between 2018 and 2020, e-cigarettes were found to aid smoking cessation in some studies^3-11^ and hinder smoking cessation in others,^12-16^ while other studies were inconclusive.^17-41^ While some studies are inconclusive on the long-term health impacts of vaping,^42-49^ others suggest that it may cause cardiovascular^50-55^ or respiratory^56-61^ diseases. In the short term, vaping e-liquids in combination with marijuana oil, often sourced informally, is potentially fatal; in the United States in 2019, 68 e-cigarette users died and over 2,800 were hospitalised with ‘e-cigarette or vaping product use associated lung injury’ (EVALI), an illness which resembles acute pneumonia.^62^

 E-cigarette use among youth is another issue that has made the use and advertising of e-cigarettes controversial. In countries such as the United States, e-cigarette use among high school students has more than doubled (from 12% to 28%) in just two years (2017-2019).^63^ The US’s e-cigarette epidemic was fuelled by the popularity of e-cigarette brands such as Juul, which dominates the global e-cigarette market with a 19% share.^2^ In the United States, Juul targeted youth with social media campaigns, a wide variety of e-liquid flavours, and a trendy, youthful brand image. Juul e-liquids also contain nicotine salts which potentiate the nicotine ‘hit’ and make the aerosol easier to inhale for first-time e-cigarette users.^63,64^ There are also concerns that e-cigarettes may act as a gateway into smoking, with a gateway effect reported in some places,^65,66^ although the evidence base for this remains inconclusive.^54,67-72^

 As of 2019, most e-cigarettes were sold in high-income Western countries where the e-cigarette industry, much of which is owned by the tobacco industry,^2^ is becoming increasingly regulated. The United States, the world’s biggest e-cigarette market, started imposing federal e-cigarette regulations in the wake of its 2019 EVALI cases.^73^ In the European Union (EU), which contains some of the world’s largest e-cigarette markets (the United Kingdom, France, Germany, Poland, Italy), e-cigarettes are partially regulated by the EU Tobacco Products Directive which came into full force in 2020.^74^ Southeast Asian countries, in contrast, have large smoking populations and undeveloped e-cigarette markets which make them desirable targets for the e-cigarette industry.^75^ Juul, which is partly owned by Altria Group (formerly Philip Morris), has also set its sights on Southeast Asian markets,^76^ with the President of Juul’s Asia-Pacific South division, in a 2019 interview, describing Asia as a “high-priority region.”^77^

 Southeast Asian countries have received relatively little attention in e-cigarette research, despite the fact that the region represents a potential area of untapped growth for the e-cigarette industry. This study aims to examine the e-cigarette situation in Southeast Asia in terms of the e-cigarette markets, advertising and promotion of e-cigarettes, reported health impacts of e-cigarette use, and policy responses in the region. We did not include heated tobacco products, such as IQOS, in our analysis. We defined Southeast Asia as the geographical region that includes Singapore, Indonesia, Malaysia, the Philippines, Myanmar, Thailand, Cambodia, Vietnam, Laos, Brunei, Taiwan, and Hong Kong. With the exception of the two Special Administrative Regions, Taiwan and Hong Kong, all of the countries are members of the Association of Southeast Asian Nations (ASEAN). We included nearby Taiwan and Hong Kong in our analysis due to their economic similarities with Singapore and Brunei, the only high-income nations in ASEAN.

## Methods

###  Market Data

 We retrieved and analyzed e-cigarette market data from the Euromonitor GMID Passport database, which included country-level data on annual e-cigarette market size from 2010-2019 and projected figures for 2020-2023, as well as country-level data on e-cigarette market shares from e-commerce. Euromonitor collects these data from company reports, customer surveys, government statistics, trade associations, trade interviews, and trade press. Data was available for all countries/territories except Laos and Myanmar.

###  Literature Search

**Figure 1:**
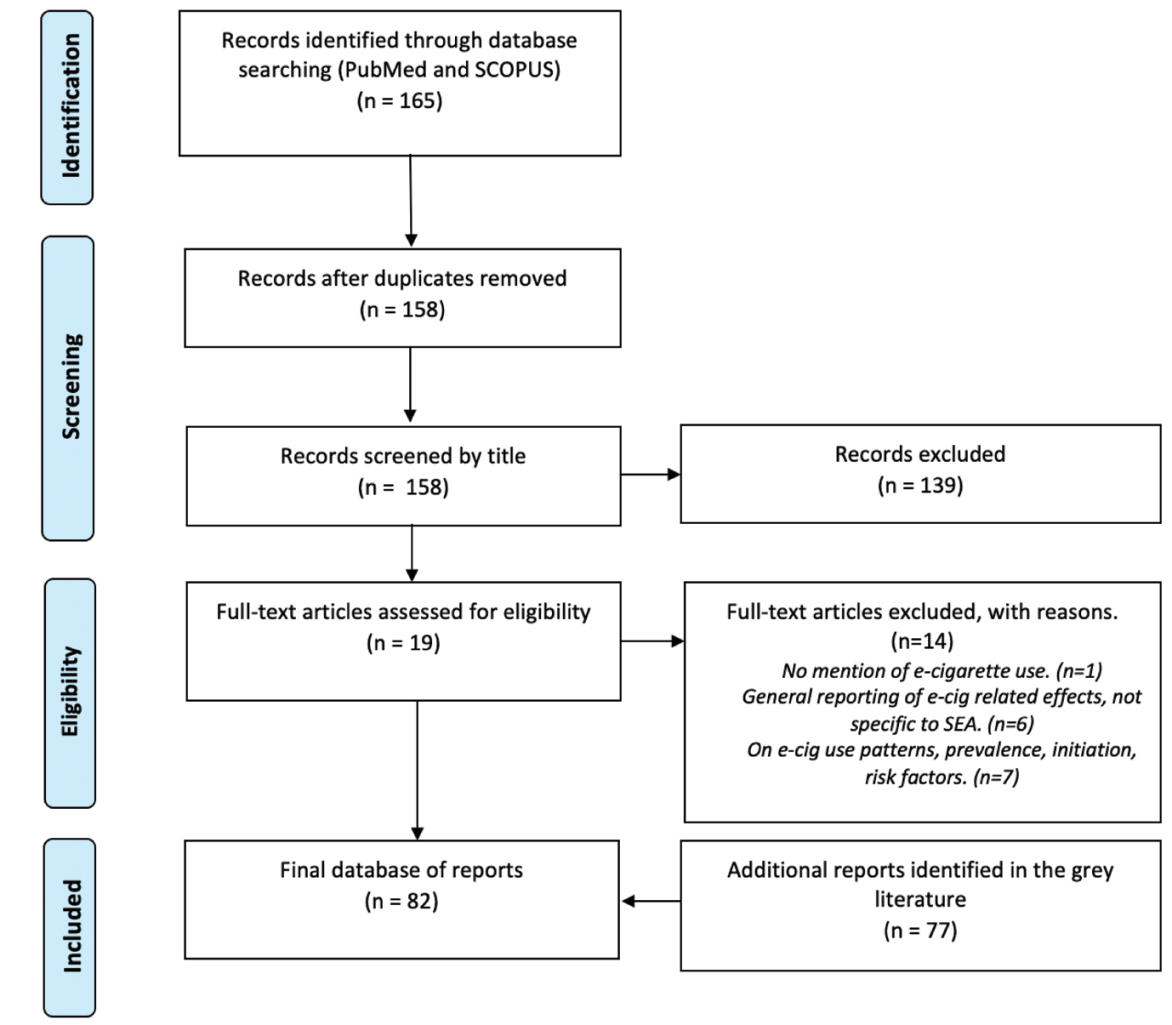
Selection Process in Literature Search. Abbreviation: SEA, Southeast Asia.

 In March-June 2020 we conducted searches in PubMed and SCOPUS for academic publications using the search string (vap* OR e-cigarette*) AND (Singapore OR Indonesia OR Malaysia OR Philippines OR Myanmar OR Thailand OR Cambodia OR Vietnam OR Lao OR Brunei OR Taiwan OR ‘Hong Kong’). Our inclusion criteria were as follows: (1) contains information on e-cigarettes, and (2) this information is specific to the Southeast Asia context. Papers that did not contain such information were excluded. The search yielded 158 papers. After title and text screening, we excluded 146 and 14 papers respectively, leaving a final set of five papers (Figure 1). Given the dearth of Southeast Asia-specific information on e-cigarettes in the academic literature, we also searched grey literature sources (Google News archives and the website of a regional tobacco control NGO) using keywords such as ‘vaping’ or ‘e-cigarette’ in combination with keywords related to location (eg, ‘Malaysia,’ ‘Singapore’). Where relevant information was found, we conducted snowball searches, in which search results inform subsequent searches, in Google or other sources (eg, government websites) as appropriate. These searches yielded a further 77 articles from government websites, media archives, and case report depositories. All relevant data were extracted and synthesized into a narrative. Details on our search strategy can be found in Supplementary file 1.

###  Online E-Cigarette Retailers

 In June 2020 we searched for online e-cigarette retailers catering to the region’s active e-cigarette markets (Malaysia, Indonesia, the Philippines, Vietnam, Taiwan, and Hong Kong). Specifically, we searched for ‘buy e-cigarette’ in the country’s respective language and Google search engine. We browsed online stores listed in the first 30 search hits for the most popular or featured e-cigarette products. We recorded the names of the most popular or featured brands and their country of origin, details of common product features, and screenshots of the online stores. For variety e-cigarette stores, we took a screenshot of the first page of products shown when searching for e-cigarettes. For specialty e-cigarette stores, we took screenshots of the store’s featured or most popular items, home page, and any e-cigarette advertisements. Further details of our search strategy can be found in Supplementary file 1.

## Results

###  Southeast Asian E-Cigarette Markets

**Figure 2:**
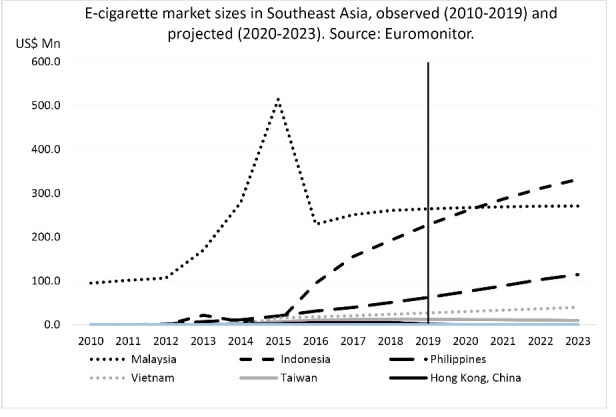
E-cigarette Market Sizes in 2010-2019 (Observed) and 2020-2023 (Projected) in Malaysia, Indonesia, the Philippines, Vietnam, Taiwan and Hong Kong.

 According to Euromonitor data (Figure 2), in 2019 e-cigarettes were sold in six Southeast Asian markets (Malaysia, Indonesia, the Philippines, Vietnam, Taiwan and Hong Kong) with a total market value of $595 million (USD). The total e-cigarette market in these places was projected to grow by 29% (to $766 million) by 2023, mainly led by growth in Indonesia and the Philippines. No e-cigarette sales were reported in Brunei, Cambodia, Singapore and Thailand, and data was not available for Laos and Myanmar.^2^

 Despite its relatively small population, Malaysia (population: 32 million) had the largest e-cigarette market among the countries listed. Its e-cigarette market grew almost five-fold in 2012-2015 (from $106 million to $514 million), before falling to less than half ($229 million) in 2016.^2^ The sharp drop was likely due to a 2015 ban on nicotine-containing e-liquids,^78^ the Malaysian National Fatwa Council declaring e-cigarettes as ‘haram’ (forbidden),^79^ and bans on vaping in the states of Penang, Kedah, Johor, Kelantan, and Terengganu in 2015-2016 covering a third of Malaysia’s population.^80^ Despite these regulatory developments, Malaysia’s e-cigarette market is projected to remain stable into the 2020’s at around $260 million per year.^2^

 The large populations of Indonesia, the Philippines and Vietnam provide ample marketing opportunities for e-cigarette companies. The e-cigarette market in Indonesia (population: 274 million) started growing steadily in 2015 and is projected to become Southeast Asia’s largest by 2021. E-cigarette markets in the Philippines (population: 109 million) and, to a lesser extent, Vietnam (population: 96 million) have been growing steadily since 2013, and are projected to continue growing into the 2020’s. Meanwhile, the e-cigarette markets of Taiwan (population: 24 million) and Hong Kong (population: 7 million) are expected to remain relatively small.^2^

###  E-Cigarette Advertising and Promotions in Southeast Asia

 While e-cigarette advertising and promotion has not been widely studied in the Southeast Asian context, a 2014 study of brick-and-mortar e-cigarette shops in Hong Kong, Malaysia, Thailand, and the Philippines found that e-cigarettes were widely promoted at the point of sale with posters, billboards, banners and pamphlets. Shops tended to take the form of stand-alone stores or booths. Rather than selling e-cigarettes alongside tobacco products, they were sold alongside common consumer products such as books, cosmetics, batteries, and hobby paraphernalia, making them appear like lifestyle products.^81^

 According to a Reuters report, prior to February 2020, when Juul announced its decision to stop all sales in Indonesia, Juul was aggressively marketing its products in the country. Juul targeted young people in Indonesia with fruit and dessert flavoured e-liquids, cinema adverts and young brand ambassadors. Through a contract with Nava Plus, an Indonesian marketing firm, the ambassadors charged 2000 rupiah ($0.15) for sample hits of Juul in trendy locations such as the Omnia Bali nightclub. Juul was promoted in shopping malls, bars, nightclubs, restaurants, convenience stores, and sleek Juul-branded stores reminiscent of Apple stores. Juul also installed kiosks at office buildings to cater to young tech employees. Juul was, as at February 2020, reportedly still selling its products in the Philippines and exploring expansion into Vietnam.^82^

**Figure 3:**
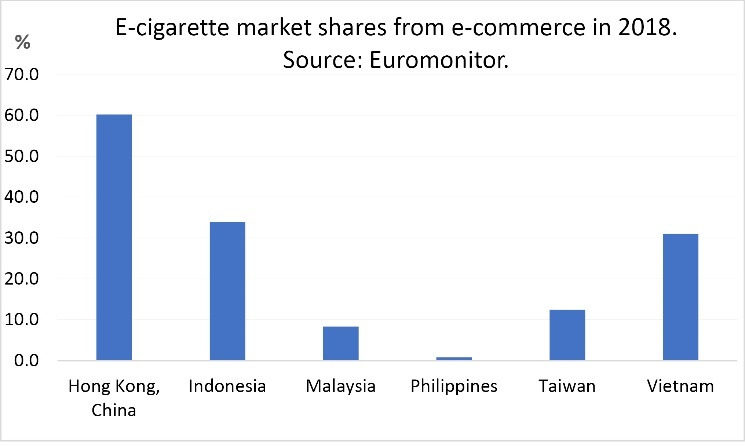
2018 E-Cigarette Market Shares From E-Commerce in Malaysia, Indonesia, the Philippines, Vietnam, Taiwan and Hong Kong.

 According to Euromonitor data (Figure 3), e-commerce is a significant and growing sales channel in the region, especially in Hong Kong, Indonesia and Vietnam.^2^ A 2014 study of online e-cigarette sales in Hong Kong, Malaysia, Thailand and the Philippines found that e-commerce sites and sites with user-generated content, such as YouTube and Reddit, were the preferred channels for promoting e-cigarettes online. In the study, the highest-ranking online shops and forums were mostly based in China for Hong Kong, and in the United States and the United Kingdom for Malaysia, Thailand and the Philippines. The e-commerce sites promoted e-cigarette devices and e-liquids with price cuts, coupons, wide varieties of flavours, and user testimonials.^81^

 In June 2020, we browsed online e-cigarette retailers catering to the six active e-cigarette markets in the region (Malaysia, Indonesia, the Philippines, Vietnam, Taiwan and Hong Kong). In Malaysia, Indonesia and the Philippines, e-cigarettes were widely available in general e-commerce stores selling a wide range of goods, as well as e-cigarette specialty shops, while in Vietnam, Taiwan and Hong Kong, e-cigarettes were sold mainly by specialty shops. In all six countries the vast majority of the popular or featured brands were from China, with a smaller number from Korea, Malaysia, Taiwan, the United States, and the United Kingdom (Table 1). Brands well-known in Western countries, such as Juul, Vuse, Blu, and MarkTen, featured rarely or not at all. These differences reflect the nascency of the industry in the region and underscore that there is room for the transnationals to compete.

**Table 1:** Name and Country of the Most Popular or Featured Brands in E-Commerce Stores

**Brand Name**	**Country of Origin**
Aleader, Artery, Aspire, Asvape, Augvape, Digiflavor, Dovpo, Eleaf, Freemax, Geek Vape, Hellvape, Hotcig, iJoy, Joyetech, KangerTech, Lost Vape, Mimo, Oukitel, Ovanty, Oxva, Rincoe, Sigelei, Sikary, Smoant, SMOK, Snowwolf, SX Mini, Teslacigs, Thinkvape, Ukeri, Uwell, Vandy Vape, Vapefly, Vaporesso, Vaporstorm, Vladdin, VooPoo, Wismec	China
Asmodus, dotMod, Hexohm, Juul, Vgod, Volta	USA
Nanostix, Relx, Rev Tech	UK
JustFog, Lil	Korea
NCIG	Malaysia
Suorin	Taiwan

 Screenshots from these websites (Supplementary file 2) illustrate the wide variety of e-cigarette designs sold in the region, often resembling items such as pens, perfume bottles, flash drives and small tech gadgets. E-liquids were sold in a wide variety of flavours, most commonly fruits, desserts (eg, custard, cheesecake) and beverages (eg, caramel macchiato, creamy lychee), although tobacco flavours (eg, Marlboro, Cuban Cigar) were also available. E-liquids also catered to the region’s tastes, with flavours such as ‘boba milk.’ The products were marketed with price promotions and user ratings. Although most websites did not feature specific adverts, an online specialty store in the Philippines featured images of a young female model vaping on its front page (Supplementary file 2), suggesting that young females are a target group.

###  Reports of E-Cigarette Related Health Effects in Southeast Asia

 While it is difficult to establish the number of cases of e-cigarette related disease or injuries in Southeast Asia due to a lack of academic research, under-reporting and uncertainties regarding disease aetiology, several cases of diseases and injuries potentially related to e-cigarette use have been reported in the region.

 Malaysian newspapers reported three suspected cases of vaping-related illness in October and November 2019. The first two cases were men who had switched from traditional cigarettes to e-cigarettes. One had developed fluid in his lungs after using a water-based e-cigarette for two weeks,^83^ while the other developed a nose tumour after using a Uwell Caliburn e-cigarette for three months.^84^ The third case was a 17-year-old boy with asthma who developed severe internal lung damage, rhabdomyolysis and kidney failure after using an e-cigarette for two weeks.^85^ News reports also detailed two possible cases of EVALI. The Philippines reported its first case of EVALI in November 2019, a 16-year-old girl who was hospitalised after a sudden onset of breathing difficulties. She had used e-cigarettes for four months, and subsequently became a dual user of cigarettes and e-cigarettes for two months before she was hospitalised.^86^ That same month in Thailand, a 48-year-old lung cancer patient was reportedly struck with EVALI after smoking an e-cigarette with marijuana.^87^

 In Hong Kong, a 2012-2013 survey of e-cigarette and tobacco use in 45 128 high school students age 12-17 found that e-cigarette users had a higher prevalence of respiratory symptoms, such as cough and phlegm, regardless of their smoking status.^88^ Irritation to the eyes, nose and upper respiratory tract associated with e-cigarette use were reported by respondents in a survey among e-cigarette users in Surabaya, Indonesia.^89^

 Several cases of accidents and injuries caused by e-cigarette explosions were reported in the news in the Philippines, Malaysia, Indonesia and Brunei in 2015-2018. In the Philippines, three cases of e-cigarette explosions were reported,^90-92^ including an e-cigarette battery explosion inside a shopping mall which caused a stampede that injured ten people.^91^ In Malaysia, three e-cigarette related injuries were reported,^93-95^ and in Indonesia, a middle aged man was reportedly hospitalised with burns to his chest, eyelids, and fingers after his e-cigarette exploded during use.^96^ In Brunei, two cases of electrical fire in buildings caused by e-cigarette explosions were reported.^97^

 Academic literature on the effects of e-cigarette use on smoking initiation and quitting in Southeast Asia was limited, generating mixed results. Taiwanese studies reported a prospective association between e-cigarette use and smoking initiation,^98^ as well as e-cigarette popularity and decreased smoking among adolescents.^99^ In Malaysia, a higher susceptibility to smoking among young people was correlated to ever-use of e-cigarettes.^100^ E-cigarettes were correlated to quit attempts in Taiwan,^101^ while e-cigarette use did not predict abstinence in Hong Kong.^102^

###  Policy Responses in Southeast Asia

 Southeast Asian countries vary considerably in terms of their e-cigarette policy responses, ranging from strict bans to no or partial regulations (Table 2).

**Table 2:** E-Cigarette Regulations in Southeast Asian Countries/Territories as at June 2020

**Country**	**Status of E-Cigarette Regulation**
Singapore	Ban on import, sale, use and possession
Cambodia	Ban on import, sale and use
Laos	Ban on import, sale and use
Brunei	Ban on import and sale; use banned in no smoking areas
Thailand	Ban on import and sale; use banned in no smoking areas
Taiwan	Partial ban on import and sale
The Philippines	Import, sale and use permitted; restrictions on advertising, flavours, use in public places and youth access (minimum legal age of 21); health warnings; taxes; ban on sale in city of Balanga
Malaysia	Import, sale and use permitted; restrictions on sale of nicotine-containing e-liquids; ban on sale in provinces of Penang, Kedah, Johor, Kelantan, and Terengganu
Hong Kong	Import, sale and use permitted; restrictions on sale of nicotine-containing e-liquids; use banned in no smoking areas; proposed ban on import and sale
Vietnam	Import, sale and use permitted; some restrictions on advertising, youth access, and use in public places
Indonesia	No specific regulations
Myanmar	No specific regulations

 The countries also differ in terms of their regulatory approach. In Singapore, Brunei and Thailand, e-cigarettes were pre-emptively classified as imitation tobacco products. These regulations were subsequently tightened, with additional laws covering the import, sale and distribution of e-cigarettes.^103^ Cambodia and Laos, in contrast, did not have an existing regulation covering e-cigarettes but issued notices to ban e-cigarette import, sale and use in 2014 (Cambodia) and 2018 (Laos).^104^ Taiwan has no blanket ban on e-cigarettes as a predefined product category, but regulates e-cigarettes under its Pharmaceutical Affairs Act. Only e-cigarettes that meet Pharmaceutical Affairs Act standards for medicines or medical devices can receive approval for import and sale in Taiwan.^105^ In May 2020, Taiwan proposed to add a category for ‘cigarette-like products’ to its tobacco legislation to ban their import, sale, use and advertising.^106^

 Singapore’s e-cigarette policy, which also bans e-cigarette purchase and possession, is the strictest in Southeast Asia. It evolved in parallel to the developing global e-cigarette market and evidence base. Singapore’s Tobacco (Control of Advertisements and Sale) Act (TCASA), first introduced in 1993 to regulate tobacco advertising and sale, was amended in 2010 to include e-cigarettes as a pre-emptive step. At the time, TCASA defined them as products believed by the Minister of Health to promote smoking or harm public health.^107,108^ In 2015,^109^ 2016,^110^ and 2017,^111^ TCASA was progressively amended to broaden its definition of e-cigarettes. The 2016 amendment covered products and components that, when assembled, may be used in a manner that mimics the act of smoking.^110^ The 2017 amendment extended the punitive measures of TCASA to also cover e-cigarette possession, purchase and use.^111^

 Southeast Asian countries/territories that regulate but permit e-cigarette use include Vietnam, Malaysia, Hong Kong and the Philippines. In Vietnam, e-cigarettes are classified as tobacco products under its tobacco control law which restricts some forms of advertising, youth access, and use in no smoking areas.^104^ In Malaysia and Hong Kong, e-cigarettes containing nicotine are covered by the countries’ poisons regulations which restrict their sale to licensed medical dealers.^103^ In Hong Kong, e-cigarettes are also covered by the Smoking (Public Health) Ordinance, which prohibits the use of e-cigarettes in no smoking areas.^112^ Both Malaysia and Hong Kong submitted bills in 2019 to tighten their e-cigarette regulations.^113,114^ Although Malaysia has yet to adopt a blanket regulation on e-cigarettes, five Malaysian states (Penang, Kedah, Johor, Kelantan and Terengganu) have banned e-cigarette sales.^80^

 The Philippines started regulating e-cigarettes in 2014 with its ‘E-cigarettes or Vape Regulation Act’ which restricted e-cigarette advertising, flavours and youth access, and mandated health warnings and the registration of e-cigarette manufacturers operating in the country. Its 2014 ‘Rules and Regulations on Electronic Nicotine Delivery System or Electronic Cigarettes’ further stipulated that e-cigarettes were to be regulated as medicinal products under the jurisdiction of the Food and Drug Administration.^103^ In February 2020, three months after the Philippines reported its first case of EVALI, the Philippine Government signed an executive order to expand the nationwide no smoking areas to include vaping and to regulate the manufacture, import, sale and marketing of unregistered e-cigarettes.^115-117^

## Discussion

 Southeast Asia’s contrasting policy responses have resulted in very different e-cigarette market scenarios. The pre-emptive responses of Singapore, Thailand, Brunei and Cambodia have likely prevented e-cigarette use in these countries. Early regulatory action in the Philippines has controlled e-cigarette use to some extent, resulting in a lower per capita consumption than in Malaysia or Indonesia, but its e-cigarette market continues to grow suggesting that its regulatory approach, based on discouraging e-cigarette use, is insufficient. Meanwhile, weak regulations have facilitated the entrenchment of e-cigarette use in Malaysian society. A similar situation is likely to transpire in Indonesia, set to become the region’s largest e-cigarette market by 2021, as well as in Vietnam and the Philippines, where e-cigarette markets are growing. These countries, with their weak regulations, large population sizes and high smoking rates, are desirable targets for e-cigarette transnationals.

 While Southeast Asia’s e-cigarette industry is still dominated by regional companies, this could change if e-cigarette transnationals such as Juul start investing more heavily in the region. In the 1980s, as trade opened up to Asian countries, tobacco transnationals started targeting Southeast Asia, especially populous countries with weak tobacco regulations such as Indonesia, the Philippines, Vietnam and Malaysia. The transnationals acquired domestic companies, targeted youth with sophisticated marketing tactics, and lobbied against regulation. As a result, Southeast Asia’ smoking rates are now among the highest globally with an average male smoking prevalence of 42%.^118^ A similar scenario may develop with e-cigarettes if the countries fail to establish e-cigarette regulations at an early stage.

 We also observed heavy targeting of youth in the region with trendy vape pen designs, a wide variety of e-liquid flavours, point of sale promotions, and adverts featuring young female models. The targeting of young people, especially females, is inconsistent with the claim that e-cigarettes are intended to help current smokers quit, especially given the low female smoking prevalence in Southeast Asia.^119^ It is also a significant public health concern given the data from international studies which indicate the potential risks for chronic health conditions or acute diseases such as EVALI, and uncertainties regarding the efficacy of e-cigarettes as a smoking cessation aid.

 The widespread use of regional e-commerce platforms for selling e-cigarettes may create additional challenges in curbing e-cigarette use among minors,^120^ and preventing cross-border e-cigarette promotions on social media.^121^ Even in countries with stricter e-cigarette regulations, such as Taiwan^122^ and Singapore,^123,124^ young people have purchased e-cigarettes from online shops and social media channels such as Instagram. This indicates that, besides regulating the purchase and possession of e-cigarettes, enforcement mechanisms are also necessary to prevent youth from purchasing e-cigarettes online. This will likely require cooperation between the countries, given the cross-border nature of illicit e-cigarette sales and promotions in the region.^123,124^

###  Limitations

 Due to the scarcity of academic research in the region, we had to rely heavily on grey literature such as news reports, which are less rigorous than peer-reviewed academic studies. This study used information from English-language academic and grey literature; thus, information may be limited where e-cigarettes have not been the subject of local research or media coverage in the English language. Data to compare the prevalence of e-cigarette use between Southeast Asian countries was not available; hence we used e-cigarette market volume data. For our market data analysis, we used the Euromonitor GMID Passport database as this was our only available source which allow comparison between the different countries. The data covered all countries/territories except Laos and Myanmar; hence we were unable to examine the e-cigarette markets of these countries. It must also be noted that, in 2019, Euromonitor received funding from the Foundation for a Smokefree World and PMI Impact, both of which are funded solely by Philip Morris International. This funding was for projects on illicit trade and a ‘Smokefree Index.’^125^ Our study did not use data from these two projects; nevertheless, Euromonitor data on the tobacco industry should be interpreted with caution.

## Conclusion

 Although Southeast Asia’s e-cigarette market is still nascent and dominated by regional companies, this could change if e-cigarette transnationals start investing more heavily in the region. Populous countries with weak e-cigarette regulations, such as Indonesia, Malaysia, Vietnam and the Philippines, are especially desirable targets for the transnationals. In these countries, e-cigarette companies are already targeting young people with a wide variety of attractive vaping devices, e-liquids and point of sale promotions. These countries should regulate e-cigarettes before their use becomes entrenched into their societies, especially among the younger generation.

## Ethical issues

 No human or animal subjects or materials were used in the study.

## Competing interests

 Authors declare that they have no competing interests.

## Authors’ contributions

 YV: conception, design, writing. GTPP, SEO: data analysis. GTLX, DL, DZ, and LMS: data acquisition. CKS: conception, design.

## Supplementary files

Supplementary file 1. Details of Searches Conducted in the Academic and Grey Literature and for Online E-Cigarette Stores.

Supplementary file 2. Screenshots From E-Cigarette Retailers Catering to Malaysia, Indonesia, Vietnam, Hong Kong, Taiwan and the Philippines.

## References

[1] World Health Organization (WHO). Electronic Nicotine Delivery Systems and Electronic Non-Nicotine Delivery Systems (ENDS/ENNDS). WHO; 2017.

[2] Euromonitor. GMID Passport Database 2020.

[3] Hajek P, Phillips-Waller A, Przulj D (2019). A randomized trial of e-cigarettes versus nicotine-replacement therapy. N Engl J Med.

[4] Lee SM, Tenney R, Wallace AW, Arjomandi M (2018). E-cigarettes versus nicotine patches for perioperative smoking cessation: a pilot randomized trial. PeerJ.

[5] Walker N, Parag V, Verbiest M, Laking G, Laugesen M, Bullen C (2020). Nicotine patches used in combination with e-cigarettes (with and without nicotine) for smoking cessation: a pragmatic, randomised trial. Lancet Respir Med.

[6] Levy DT, Yuan Z, Li Y, Alberg AJ, Cummings KM (2019). A modeling approach to gauging the effects of nicotine vaping product use on cessation from cigarettes: what do we know, what do we need to know?. Addiction.

[7] Qin Y, Edjoc R, Osgood ND. Effect of e-cigarette use and social network on smoking behavior change: an agent-based model of e-cigarette and cigarette interaction. Social, Cultural, and Behavioral Modeling: 12th International Conference, SBP-BRiMS 2019. Washington, DC, USA: Springer International; 2019.

[8] Warner KE, Mendez D (2019). E-cigarettes: comparing the possible risks of increasing smoking initiation with the potential benefits of increasing smoking cessation. Nicotine Tob Res.

[9] Farsalinos K, Siakas G, Poulas K, Voudris V, Merakou K, Barbouni A (2019). E-cigarette use is strongly associated with recent smoking cessation: an analysis of a representative population sample in Greece. Intern Emerg Med.

[10] Kalkhoran S, Chang Y, Rigotti NA (2019). E-cigarettes and smoking cessation in smokers with chronic conditions. Am J Prev Med.

[11] Villanti AC, Feirman SP, Niaura RS (2018). How do we determine the impact of e-cigarettes on cigarette smoking cessation or reduction? review and recommendations for answering the research question with scientific rigor. Addiction.

[12] Skerry A, Lusher J, Banbury S (2018). Electronic cigarette users lack intention to quit vaping. MOJ Addict Med Ther.

[13] Subialka Nowariak EN, Lien RK, Boyle RG, Amato MS, Beebe LA (2018). E-cigarette use among treatment-seeking smokers: moderation of abstinence by use frequency. Addict Behav.

[14] Brose LS, Bowen J, McNeill A, Partos TR (2019). Associations between vaping and relapse to smoking: preliminary findings from a longitudinal survey in the UK. Harm Reduct J.

[15] Dai H, Leventhal AM (2019). Association of electronic cigarette vaping and subsequent smoking relapse among former smokers. Drug Alcohol Depend.

[16] Kulik MC, Lisha NE, Glantz SA (2018). E-cigarettes associated with depressed smoking cessation: a cross-sectional study of 28 European Union countries. Am J Prev Med.

[17] Diemert L, Bayoumy D, Pelletier H, Schwartz R, O’Connor S. E-Cigarette Use for Smoking Cessation: Scientific Evidence and Smokers’ Experiences. Toronto, CA: Ontario Tobacco Research Unit; 2019.

[18] Erku D, Gartner CE, Morphett K, Snoswell CL, Steadman KJ (2020). Nicotine vaping products as a harm reduction tool among smokers: Review of evidence and implications for pharmacy practice. Res Social Adm Pharm.

[19] Farsalinos K. Electronic cigarettes: an aid in smoking cessation, or a new health hazard? Ther Adv Respir Dis 2018;12. 10.1177/1753465817744960. PMC5937152 29214890

[20] Franks AS, Sando K, McBane S (2018). Do electronic cigarettes have a role in tobacco cessation?. Pharmacotherapy.

[21] Gentry S, Forouhi NG, Notley C (2019). Are electronic cigarettes an effective aid to smoking cessation or reduction among vulnerable groups? a systematic review of quantitative and qualitative evidence. Nicotine Tob Res.

[22] Hartmann-Boyce J, Begh R, Aveyard P (2018). Electronic cigarettes for smoking cessation. BMJ.

[23] Patil S, Arakeri G, Patil S (2020). Are electronic nicotine delivery systems (ENDs) helping cigarette smokers quit?-current evidence. J Oral Pathol Med.

[24] Whitehouse E, Lai J, Golub JE, Farley JE (2018). A systematic review of the effectiveness of smoking cessation interventions among patients with tuberculosis. Public Health Action.

[25] Wolf S, O’Sullivan S, Dean R, Owens T (2019). Does utilization of electronic cigarettes facilitate smoking cessation compared to other interventions?. J Okla State Med Assoc.

[26] Worku D, Worku E (2019). A narrative review evaluating the safety and efficacy of e-cigarettes as a newly marketed smoking cessation tool. SAGE Open Med.

[27] Chiang SC, Abroms LC, Cleary SD, Pant I, Doherty L, Krishnan N (2019). E-cigarettes and smoking cessation: a prospective study of a national sample of pregnant smokers. BMC Public Health.

[28] uillaumier A, Manning V, Wynne O (2018). Electronic nicotine devices to aid smoking cessation by alcohol- and drug-dependent clients: protocol for a pilot randomised controlled trial. Trials.

[29] Halpern SD, Harhay MO, Saulsgiver K, Brophy C, Troxel AB, Volpp KG (2018). A pragmatic trial of e-cigarettes, incentives, and drugs for smoking cessation. N Engl J Med.

[30] Lee SH, Ahn SH, Cheong YS (2019). Effect of electronic cigarettes on smoking reduction and cessation in Korean male smokers: a randomized controlled study. J Am Board Fam Med.

[31] Berry KM, Reynolds LM, Collins JM (2019). E-cigarette initiation and associated changes in smoking cessation and reduction: the Population Assessment of Tobacco and Health Study, 2013-2015. Tob Control.

[32] Chen JC (2018). Flavored e-cigarette use and cigarette smoking reduction and cessation-a large national study among young adult smokers. Subst Use Misuse.

[33] Watkins SL, Thrul J, Max W, Ling PM (2020). Real-world effectiveness of smoking cessation strategies for young and older adults: findings from a nationally representative cohort. Nicotine Tob Res.

[34] Gomajee R, El-Khoury F, Goldberg M (2019). Electronic cigarette use and smoking reduction–longitudinal data from CONSTANCES cohort study. Eur J Public Health.

[35] Jackson SE, Shahab L, West R, Brown J (2020). Associations between dual use of e-cigarettes and smoking cessation: a prospective study of smokers in England. Addict Behav.

[36] Lozano P, Arillo-Santillán E, Barrientos-Gutiérrez I, Zavala-Arciniega L, Reynales-Shigematsu LM, Thrasher JF (2019). E-cigarette use and its association with smoking reduction and cessation intentions among Mexican smokers. Salud Publica Mex.

[37] Brandon KO, Simmons VN, Meltzer LR (2019). Vaping characteristics and expectancies are associated with smoking cessation propensity among dual users of combustible and electronic cigarettes. Addiction.

[38] Browne M, Todd DG (2018). Then and now: consumption and dependence in e-cigarette users who formerly smoked cigarettes. Addict Behav.

[39] Foong AL, Lai MY (2018). E-cigarettes for smoking cessation: why do users continue with e-cigarettes?. Asian Soc Sci.

[40] Hsu G, Gamst AC, Zhuang YL, Wolfson T, Zhu SH (2019). A comparison of e-cigarette use patterns and smoking cessation behavior among vapers by primary place of purchase. Int J Environ Res Public Health.

[41] Notley C, Ward E, Dawkins L, Holland R (2018). The unique contribution of e-cigarettes for tobacco harm reduction in supporting smoking relapse prevention. Harm Reduct J.

[42] Biondi-Zoccai G, Sciarretta S, Bullen C (2019). Acute effects of heat-not-burn, electronic vaping, and traditional tobacco combustion cigarettes: the Sapienza university of Rome-vascular assessment of proatherosclerotic effects of smoking (SUR-VAPES) 2 randomized trial. J Am Heart Assoc.

[43] Chaumont M, de Becker B, Zaher W (2018). Differential effects of e-cigarette on microvascular endothelial function, arterial stiffness and oxidative stress: a randomized crossover trial. Sci Rep.

[44] Eltorai AE, Choi AR, Eltorai AS (2019). Impact of electronic cigarettes on various organ systems. Respir Care.

[45] Gotts JE, Jordt SE, McConnell R, Tarran R (2019). What are the respiratory effects of e-cigarettes?. BMJ.

[46] Kaur G, Pinkston R, McLemore B, Dorsey WC, Batra S (2018). Immunological and toxicological risk assessment of e-cigarettes. Eur Respir Rev.

[47] Papaefstathiou E, Stylianou M, Agapiou A (2019). Main and side stream effects of electronic cigarettes. J Environ Manage.

[48] Ratajczak A, Feleszko W, Smith DM, Goniewicz M (2018). How close are we to definitively identifying the respiratory health effects of e-cigarettes?. Expert Rev Respir Med.

[49] Thirión-Romero I, Pérez-Padilla R, Zabert G, Barrientos-Gutiérrez I (2019). Respiratory impact of electronic cigarettes and “low-risk” tobacco. Rev Invest Clin.

[50] Franzen KF, Willig J, Cayo Talavera S (2018). E-cigarettes and cigarettes worsen peripheral and central hemodynamics as well as arterial stiffness: a randomized, double-blinded pilot study. Vasc Med.

[51] Osei AD, Mirbolouk M, Orimoloye OA (2019). Association between e-cigarette use and cardiovascular disease among never and current combustible-cigarette smokers. Am J Med.

[52] Darville A, Hahn EJ (2019). E-cigarettes and atherosclerotic cardiovascular disease: what clinicians and researchers need to know. Curr Atheroscler Rep.

[53] MacDonald A, Middlekauff HR (2019). Electronic cigarettes and cardiovascular health: what do we know so far?. Vasc Health Risk Manag.

[54] Alzahrani T, Pena I, Temesgen N, Glantz SA (2018). Association between electronic cigarette use and myocardial infarction. Am J Prev Med.

[55] Ikonomidis I, Vlastos D, Kourea K (2018). Electronic cigarette smoking increases arterial stiffness and oxidative stress to a lesser extent than a single conventional cigarette: an acute and chronic study. Circulation.

[56] Blagev DP, Harris D, Dunn AC, Guidry DW, Grissom CK, Lanspa MJ (2019). Clinical presentation, treatment, and short-term outcomes of lung injury associated with e-cigarettes or vaping: a prospective observational cohort study. Lancet.

[57] Chaumont M, van de Borne P, Bernard A (2019). Fourth generation e-cigarette vaping induces transient lung inflammation and gas exchange disturbances: results from two randomized clinical trials. Am J Physiol Lung Cell Mol Physiol.

[58] Higham A, Bostock D, Booth G, Dungwa JV, Singh D (2018). The effect of electronic cigarette and tobacco smoke exposure on COPD bronchial epithelial cell inflammatory responses. Int J Chron Obstruct Pulmon Dis.

[59] Scott A, Lugg ST, Aldridge K (2018). Pro-inflammatory effects of e-cigarette vapour condensate on human alveolar macrophages. Thorax.

[60] Miyashita L, Suri R, Dearing E (2018). E-cigarette vapour enhances pneumococcal adherence to airway epithelial cells. Eur Respir J.

[61] Ghosh A, Coakley RC, Mascenik T (2018). Chronic e-cigarette exposure alters the human bronchial epithelial proteome. Am J Respir Crit Care Med.

[62] Centers for Disease Control and Prevention (CDC). Outbreak of Lung Injury Associated with the Use of E-Cigarette, or Vaping, Products. CDC; 2020.

[63] Campaign for Tobacco-Free Kids. JUUL and Other High Nicotine E-Cigarettes Are Addicting a New Generation of Youth. United States: Campaign for Tobacco-Free Kids; 2020.

[64] Campaign for Tobacco-Free Kids. JUUL and Youth: Rising E-Cigarette Popularity. https://www.tobaccofreekids.org/assets/factsheets/0394.pdf. Accessed May 26, 2020. Published 2019.

[65] Grant JE, Lust K, Fridberg DJ, King AC, Chamberlain SR (2019). E-cigarette use (vaping) is associated with illicit drug use, mental health problems, and impulsivity in university students. Ann Clin Psychiatry.

[66] Beard E, West R, Michie S, Brown J (2020). Association of prevalence of electronic cigarette use with smoking cessation and cigarette consumption in England: a time-series analysis between 2006 and 2017. Addiction.

[67] Hallingberg B, Maynard OM, Bauld L (2020). Have e-cigarettes renormalised or displaced youth smoking? results of a segmented regression analysis of repeated cross sectional survey data in England, Scotland and Wales. Tob Control.

[68] Morgenstern M, Nies A, Goecke M, Hanewinkel R (2018). E-cigarettes and the use of conventional cigarettes. Dtsch Arztebl Int.

[69] Fadus MC, Smith TT, Squeglia LM (2019). The rise of e-cigarettes, pod mod devices, and JUUL among youth: factors influencing use, health implications, and downstream effects. Drug Alcohol Depend.

[70] Perikleous EP, Steiropoulos P, Paraskakis E, Constantinidis TC, Nena E (2018). E-cigarette use among adolescents: an overview of the literature and future perspectives. Front Public Health.

[71] Chyderiotis S, Benmarhnia T, Beck F, Spilka S, Legleye S (2020). Does e-cigarette experimentation increase the transition to daily smoking among young ever-smokers in France?. Drug Alcohol Depend.

[72] Levy DT, Borland R, Lindblom EN (2018). Potential deaths averted in USA by replacing cigarettes with e-cigarettes. Tob Control.

[73] U.S. Food and Drug Administration (FDA). Enforcement Priorities for Electronic Nicotine Delivery System (ENDS) and Other Deemed Products on the Market Without Premarket Authorization. In: Center for Tobacco Products, ed. United States: FDA; 2020.

[74] EU Trade Helpdesk. Ban on flavoured tobacco products and new requirements for e-cigarettes from 20 May 2016. https://trade.ec.europa.eu/doclib/docs/2016/july/tradoc_154817.pdf. Accessed May 10, 2020. Published July 2016.

[75] O’Donovan L. An Overview of the E-Cigarette Market. Tobacco Asia; 2017.

[76] Abad M. After U.S. Scandal, JUUL Develops New Market in the Philippines. Southeast Asia Tobacco Control Alliance (SEATCA); 2020.

[77] Du L, Einhorn B. Asia Smokers Are New Target for Embattled E-Cigarette Maker Juul. Bloomberg; 2019.

[78] Hasnan L. Malaysia Wants to Regulate E-Cigarettes. The ASEAN Post. October 20, 2019. https://theaseanpost.com/article/malaysia-wants-regulate-e-cigarettes.

[79] The Straits Times. Malaysia’s Fatwa Council Declares Electronic Cigarettes as ‘Haram’ or Forbidden. The Straits Times; 2015.

[80] The Straits Times. Malaysia’s Terengganu 5th State to Ban Vaping. The Straits Times; 2015.

[81] Southeast Asia Tobacco Control Alliance (SEATCA). Electronic Cigarettes in Asia - A Review of Promotions and Availability. Bangkok, Thailand: SEATCA; 2014.

[82] Kirkham C, Potkin F, Morales NJ. Exclusive: Juul Halts Indonesia E-Cigarette Sales, Throwing Asia Expansion in Doubt. Southeast Asia Tobacco Control Alliance (SEATCA); 2020.

[83] Sheralyn. 34yo M’sian Hospitalised for Fluid & Fungus in His Lungs After Switching to Vape for 2 Weeks. WORLD OF BUZZ. October 14, 2019. https://worldofbuzz.com/34yo-msian-hospitalised-for-fluid-fungus-in-his-lungs-after-switching-to-vape-for-2-weeks/.

[84] Iman K. Malaysia Just Got its First Case of Vape-Related Illness. So How Unsafe is Vaping? CILISOS; 2019.

[85] New Straits Times. Mysterious Vaping-Related Illness Surfaces in Malaysia. New Straits Times. November 8, 2019. https://www.nst.com.my/news/nation/2019/11/536961/mysterious-vaping-related-illness-surfaces-malaysia.

[86] The Nation. DOH Cites 1st PH Case of Vape-Related Injury. The Nation. 2019.

[87] Coconuts Bangkok. Marijuana Vaping Disease Strikes Thai Patient: Doctor. Coconuts Bangkok; 2019.

[88] Wang MP, Ho SY, Leung LT, Lam TH (2016). Electronic cigarette use and respiratory symptoms in Chinese adolescents in Hong Kong. JAMA Pediatr.

[89] Lestari KS, Humairo MV, Agustina U (2018). Formaldehyde vapor concentration in electronic cigarettes and health complaints of electronic cigarettes smokers in Indonesia. J Environ Public Health.

[90] Nelz J. Vape Explodes While Filipino Testing Out the Product at Shop. Philippine News. January 16, 2017. https://philnews.ph/2017/01/16/viral-vape-explodes-filipino-testing-product-shop/.

[91] Flora IO. 10 Injured in Pampanga Mall Stampede. SunStar; 2018.

[92] de Vera A. DOH to Probe Alleged Explosion of Vaping Device Wounding Face of 17-Year-Old Boy. Manila Bulletin; 2018.

[93] Ghazali R. Vape Battery Catches Fire in Aircraft: ‘It Was a Case of Ignorance.’ AsiaOne; 2015.

[94] Berita Harian. Man Injured After E-Cigarette Explodes in Face. The Star; 2015.

[95] Khairudin MN, Mohd Zahidin AZ, Bastion ML. Front to back ocular injury from a vaping-related explosion. BMJ Case Rep 2016;2016. 10.1136/bcr-2016-214964. PMC4840649 27048399

[96] Tribun Lampung. Rokok Elektronik yang Diisap Meledak, Dada Pria Ini Terbakar dan Kelopak Matanya Robek (‘Electronic Cigarette Explosion Caused Chest Burns and Injury to the Eye Lid’). Tribum Lampung; 2016.

[97] Mohamad L. Faulty Electrical Wiring Behind Most of House Fire Accidents. Borneo Bulletin; 2018.

[98] Chien YN, Gao W, Sanna M (2019). Electronic cigarette use and smoking initiation in Taiwan: evidence from the first prospective study in Asia. Int J Environ Res Public Health.

[99] Gao W, Sanna M, Chuluunbaatar E, Tsai MK, Levy DT, Wen CP (2021). Are e-cigarettes reviving the popularity of conventional smoking among Taiwanese male adolescents? a time-trend population-based analysis for 2004-2017. Tob Control.

[100] Lim KH, Ghazali SM, Lim HL (2019). Smoking susceptibility among non-smoking school-going adolescents in Malaysia: findings from a national school-based survey. BMJ Open.

[101] Chen PC, Chang LC, Hsu C, Lee YC (2019). Electronic cigarette use and attempts to quit smoking cigarettes among adolescents in Taiwan. J Adolesc Health.

[102] Wu SY, Wang MP, Li WH, Kwong AC, Lai VW, Lam TH (2018). Does electronic cigarette use predict abstinence from conventional cigarettes among smokers in Hong Kong?. Int J Environ Res Public Health.

[103] Jin P, Jiang JY (2017). E-cigarettes in ten Southeast Asian countries: a comparison of national regulations. Glob Health J.

[104] Global Tobacco Control. E-Cigarette Policy Scan. https://globaltobaccocontrol.org/e-cigarette/countries. Accessed May 6, 2020. Published 2020.

[105] Shih CS, Etter JF (2019). Stakeholders’ views on e-cigarette legislation: a qualitative study in Taiwan. Front Public Health.

[106] Ministry of Health and Welfare Taiwan. General information on amendments to Tobacco Hazards Prevention Act. Taiwan; 2020.

[107] Smoking (Control of Advertisements and Sale of Tobacco) (Amendment) Act 2010, No. 17 of 2010 (2010).

[108] Ministry of Health. Second Reading Speech for Proposed Tobacco (Control of Advertisements and Sale) Act. Ministry of Health; 2010.

[109] Ministry of Health. Singapore Enhances Tobacco Control Efforts with Ban on Emerging Tobacco Products. Ministry of Health; 2015.

[110] Tobacco (Control of Advertisements and Sale) (Amendment) Act 2016, No.9 of 2016 (2016).

[111] Tobacco (Control of Advertisements and Sale) (Amendment) Act 2017, No.46 of 2017 (2017).

[112] Hong Kong Legislative Council Secretariat. Regulation of e-cigarettes and heated tobacco products in selected places. In: Research Office, ed. Hong Kong, 2018.

[113] RTHK. Govt to Re-Table Vape Ban to Next Legco. RTHK; 2020.

[114] Caruana D. Malaysia Considers Total E-Cigarette Ban. Vaping Post; 2019.

[115] Prohibiting the Manufacture, Distribution, Marketing and Sale of Unregistered And/Or Adulterated Electronic Nicotine/Non-Nicotine Delivery Systems, Heated Tobacco Products and Other Novel Tobacco Prodcts, Amending Executive Order No. n26 (S, 2017) and For Other Purposes, Executive Order No. 106 (2020).

[116] CNN Philippines. Nationwide Smoking Ban Now Covers Vape. CNN Philippines. February 28, 2020. https://www.cnnphilippines.com/news/2020/2/28/duterte-vaping-ban-philippines-2020.html.

[117] Xu X. Philippines’ Duterte Orders Nationwide Ban on Public Vaping, Unregistered E-Cigarettes Sales. Xinhua. February 28, 2020. http://www.xinhuanet.com/english/2020-02/28/c_138827536.htm.

[118] Amul GGH, Tan GPP, van der Eijk Y. A systematic review of tobacco industry tactics in Southeast Asia: lessons for other low- and middle-income regions. Int J Health Policy Manag 2020; In Press. 10.34172/ijhpm.2020.97. PMC9056152 32610812

[119] Southeast Asia Tobacco Control Alliance (SEATCA). The ASEAN Tobacco Control Atlas. 4th ed. Bangkok, Thailand: SEATCA; 2020.

[120] Williams RS, Derrick J, Ribisl KM (2015). Electronic cigarette sales to minors via the internet. JAMA Pediatr.

[121] Berg CJ, Haardörfer R, Cahn Z (2019). The association between Twitter activity and e-cigarette purchasing. Tob Regul Sci.

[122] Lee IC. E-Cigarette Usage Among Young on the Rise, HPA Says. Taipei Times. August 22, 2019. https://www.taipeitimes.com/News/taiwan/archives/2019/08/22/2003720925.

[123] Lam L. Man’s Illegal Online E-Cigarette Business Derailed by HSA, Fined S$45,000. CNA. July 17, 2020. https://www.channelnewsasia.com/news/singapore/man-ran-illegal-online-e-cigarette-business-until-hsa-nabbed-him-12940564.

[124] Lim J. Ban on E-Cigarettes: 465 People Caught for Possessing Products, HSA ‘Working Closely’ with Carousell, Instagram to Monitor Sales. Today. November 5, 2019. https://www.todayonline.com/singapore/ban-e-cigarettes-465-people-caught-possessing-products-hsa-working-closely-carousell-instagram-sales.

[125] Gallagher A, Gilmore A. Euromonitor international now accepts tobacco industry funding: a WIN for PMI at the expense of research on the tobacco industry. https://blogs.bmj.com/tc/2019/04/08/euromonitor-international-now-accepts-tobacco-industry-funding-a-win-for-pmi-at-the-expense-of-research-on-the-tobacco-industry/. Posted April 8, 2019.

