# Supplementary material for: E-Cigarette Markets and Policy Responses in Southeast Asia: A Scoping Review
Source: Int J Health Policy Manag. 2021 Apr 13;11(9):1616–24. doi: 10.34172/ijhpm.2021.25 (PMC9808234; doi:10.34172/ijhpm.2021.25)
Supplement: Supplementary file 1 — Details of Searches Conducted in the Academic and Grey Literature and for Online E-Cigarette Stores. [file ijhpm-11-1616-s001.pdf]

Article title: E-Cigarette Markets and Policy Responses in Southeast Asia: A Scoping Review

Journal name: International Journal of Health Policy and Management (IJHPM)

Authors' information: Yvette van der Eijk[1]*, Grace Tan Ping Ping[1], Suan Ee Ong[1,2], Grace Tan Li Xin[3], David Li[4], Dijin Zhang[5], Loo Min Shuen[6], Chia Kee Seng[1]

[1]Saw Swee Hock School of Public Health, National University of Singapore, Singapore, Singapore.

[2]Research for Impact, Singapore, Singapore.

[3]Department of Political Science, Faculty of Arts and Social Sciences, National University of Singapore, Singapore, Singapore.

[4]Deparment of Civil and Environmental Engineering, Faculty of Engineering, University of Alberta, Edmonton, AB, Canada.

[5]Department of Biological Sciences, Faculty of Science, National University of Singapore, Singapore, Singapore.

[6]Department of Psychology, Faculty of Arts and Social Sciences, National University of Singapore, Singapore, Singapore.

(*corresponding author: yvette.eijk@nus.edu.sg)

Supplementary file 1. Details of Searches Conducted in the Academic and Grey Literature and for Online E-Cigarette Stores

**Search string/keywords**	**Database**	**Hits**	**Included**
'(vap* OR e-cigarette*) AND (Singapore OR Indonesia OR Malaysia OR Philippines OR Myanmar OR Thailand OR Cambodia OR Vietnam OR Lao OR Brunei OR Taiwan OR 'Hong Kong')'	PubMed	117	4
'(vap* OR e-cigarette*) AND (Singapore OR Indonesia OR Malaysia OR Philippines OR Myanmar OR Thailand OR Cambodia OR Vietnam OR Lao OR Brunei OR Taiwan OR 'Hong Kong')'	SCOPUS	48	1
'Vaping', 'e-cigarette' and country in question ('Singapore', 'Indonesia', 'Malaysia', 'Philippines', 'Myanmar',	Google News	225	16

'Thailand', 'Cambodia', 'Vietnam', 'Laos', 'Brunei', 'Taiwan', 'Hong Kong')			
'Vaping', 'e-cigarette'	Southeast Asian Tobacco Control Alliance website	494	61

Details of searches conducted for online e-cigarette stores.

Search details	*Variety e-commerce sites*	*Specialty e-cigarette stores*
'beli ecig', Google.com.my (Malaysia)	Area51manado.com<br>Bukalapak.com<br>Lazada.com.my<br>Shopee.com.my<br>Tokopedia.com	Geekvape.com<br>Juul.id<br>Rayvapor.com<br>Snowplustech.com<br>Thevapeshophk.com<br>Vandyvape.com<br>Vapeclubmy.com<br>Vapevandal.com<br>Wismec.com
'beli vape', Google.co.id (Indonesia)	Blibli.com<br>Bukalapak.com<br>Idpriceprice.com<br>Iprice.co.id<br>Lazada.co.id<br>My-best.id<br>Ralali.com<br>Shopee.co.id	Gearvita.id<br>Jakartarvaporshop.com<br>Rayvapor.com<br>Vapehan.com<br>Vapeoi.id<br>Vaporizerjakarta.com
'bumili vape', Google.com.ph (Philippines)	Carousell.ph<br>Iprice.ph<br>Lazada.com.ph<br>My-best.ph<br>Ph.priceprice.com<br>Shopee.ph	Ejuice.ph<br>Inthefog.ph<br>Juul.ph<br>Mcmmods.com.ph<br>Mistwoodvape.com<br>Myvape.com.ph<br>Ph.relxnow.com

		Planetvape.ph<br>Smoktech.com<br>Vapemnl.com<br>Vapephil.com
‘Mua thuốc lá điện tử ở đâu’, Google.com.vn (Vietnam)		Likevape.vn<br>Shishadientu.net<br>Shophanghieu.top<br>Vapechinhhang.com<br>Vapepro.vn<br>Vapesaigon.com<br>Vaporshop.com.vn
‘購買電子煙台灣’, Google.com.tw (Taiwan)		Bacc.tw<br>Find.ruten.com.tw<br>Vape104.com<br>Vapebullhk.com<br>Zero18.com
‘購買電子煙香港’, Google.com.hk (Hong Kong)		Heaterhk.com<br>Iyes.hk<br>Relxhk.com<br>Vapebullhk.com<br>Vaporclub.hk
