# Supplementary material for: E-Cigarette Markets and Policy Responses in Southeast Asia: A Scoping Review
Source: Int J Health Policy Manag. 2021 Apr 13;11(9):1616–24. doi: 10.34172/ijhpm.2021.25 (PMC9808234; doi:10.34172/ijhpm.2021.25)
Supplement: Supplementary file 2 — Screenshots From E-Cigarette Retailers Catering to Malaysia, Indonesia, Vietnam, Hong Kong, Taiwan and the Philippines. [file ijhpm-11-1616-s002.pdf]

Article title: E-Cigarette Markets and Policy Responses in Southeast Asia: A Scoping Review

Journal name: International Journal of Health Policy and Management (IJHPM)

Authors' information: Yvette van der Eijk[1]*, Grace Tan Ping Ping[1], Suan Ee Ong[1,2], Grace Tan Li Xin[3], David Li[4], Dijin Zhang[5], Loo Min Shuen[6], Chia Kee Seng[1]

[1]Saw Swee Hock School of Public Health, National University of Singapore, Singapore, Singapore.

[2]Research for Impact, Singapore, Singapore.

[3]Department of Political Science, Faculty of Arts and Social Sciences, National University of Singapore, Singapore, Singapore.

[4]Deparment of Civil and Environmental Engineering, Faculty of Engineering, University of Alberta, Edmonton, AB, Canada.

[5]Department of Biological Sciences, Faculty of Science, National University of Singapore, Singapore, Singapore.

[6]Department of Psychology, Faculty of Arts and Social Sciences, National University of Singapore, Singapore, Singapore.

(*corresponding author: yvette.eijk@nus.edu.sg)

Supplementary file 2. Screenshots From E-Cigarette Retailers Catering to Malaysia, Indonesia, Vietnam, Hong Kong, Taiwan and the Philippines.

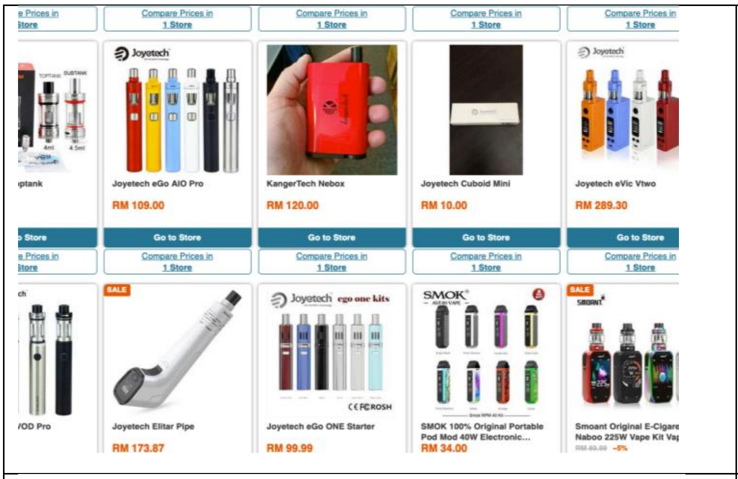
Compare Prices in 1 Store
Joyetech eGo AIO Pro
RM 109.00
KangerTech Nebox
RM 120.00
Joyetech Cuboid Mini
RM 10.00
Joyetech eVic Vtwo
RM 289.30
Go to Store
Joyetech Elitar Pipe
RM 173.87
Joyetech eGo ONE Starter
RM 99.99
SMOK 100% Original Portable Pod Mod 40W Electronic...
RM 34.00
Smoant Original E-Cigare Naboo 225W Vape Kit Vap

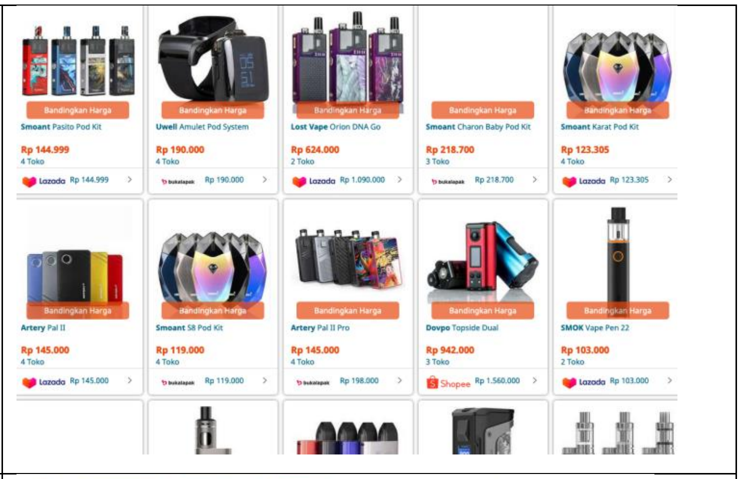
Bandingkan Harga
Smoant Pasito Pod Kit
Rp 144.999
Uwell Amulet Pod System
Rp 190.000
Lost Vape Orion DNA Go
Rp 624.000
Smoant Charon Baby Pod Kit
Rp 218.700
Smoant Karat Pod Kit
Rp 123.305
Artery Pal II
Rp 145.000
Smoant S8 Pod Kit
Rp 119.000
Artery Pal II Pro
Rp 145.000
Dovpo Topside Dual
Rp 942.000
SMOK Vape Pen 22
Rp 103.000

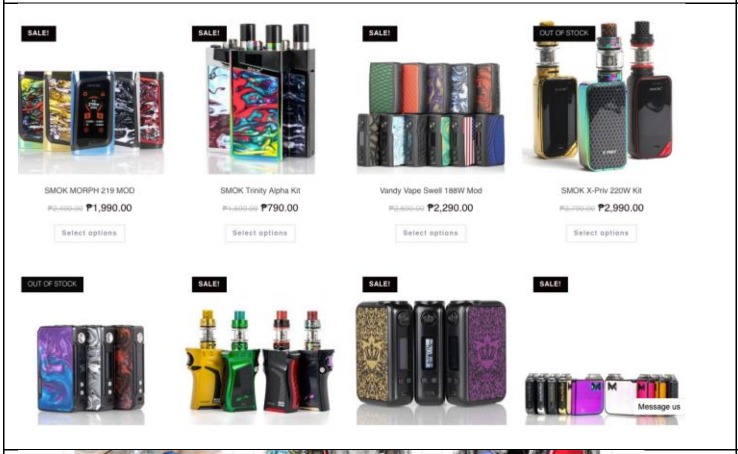
SALE!
SMOK MORPH 219 MOD
₱1,990.00
SMOK Trinity Alpha Kit
₱790.00
Vandy Vape Swell 188W Mod
₱2,290.00
OUT OF STOCK
SMOK X-Priv 220W Kit
₱2,990.00
Select options

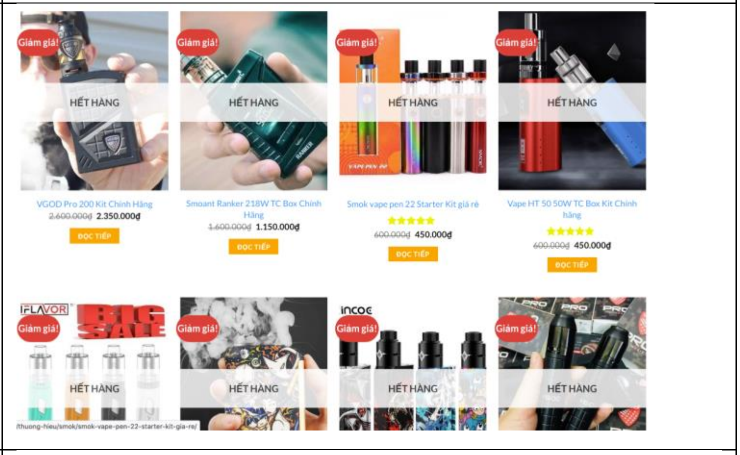
Giảm giá!
HẾT HÀNG
VGOD Pro 200 Kit Chính Hãng
2.350.000₫
Smoant Ranker 218W TC Box Chính Hãng
1.150.000₫
Smok vape pen 22 Starter Kit giá rẻ
450.000₫
Vape HT 50 50W TC Box Kit Chính hãng
450.000₫
ĐỌC TIẾP

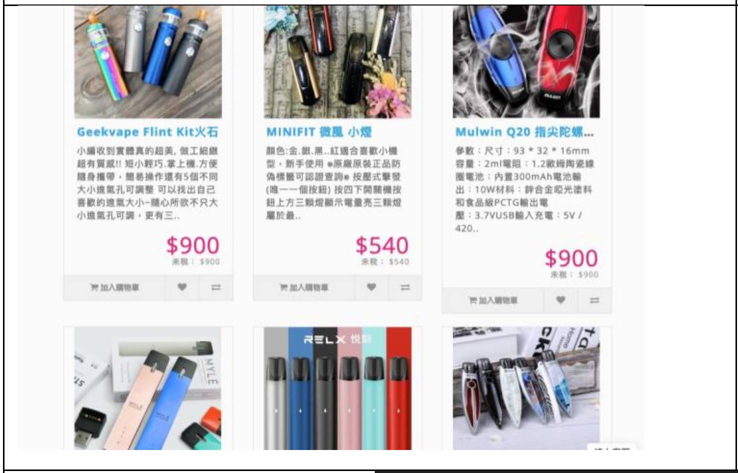
Geekvape Flint Kit火石
MINIFIT 微風 小煙
Mulwin Q20 指尖陀螺...
$900
$540
$900

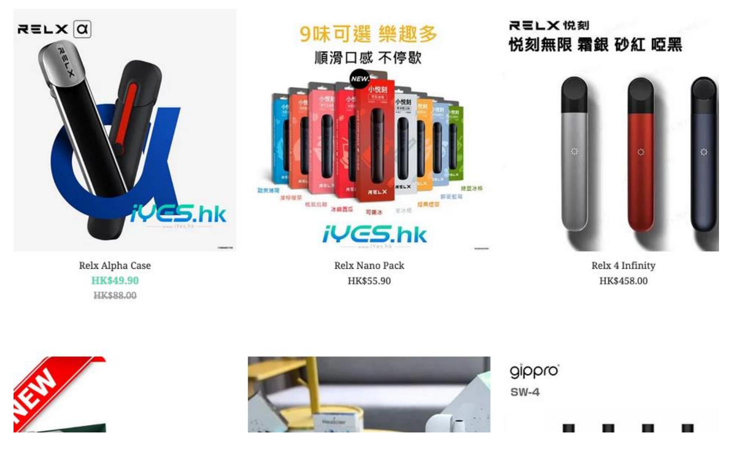
RELX
9味可選 樂趣多
順滑口感 不停歇
RELX 悅刻
悅刻無限 霜銀 砂紅 啞黑
Relx Alpha Case
HK$49.90
HK$88.00
Relx Nano Pack
HK$55.90
Relx 4 Infinity
HK$458.00
gippro
SW-4

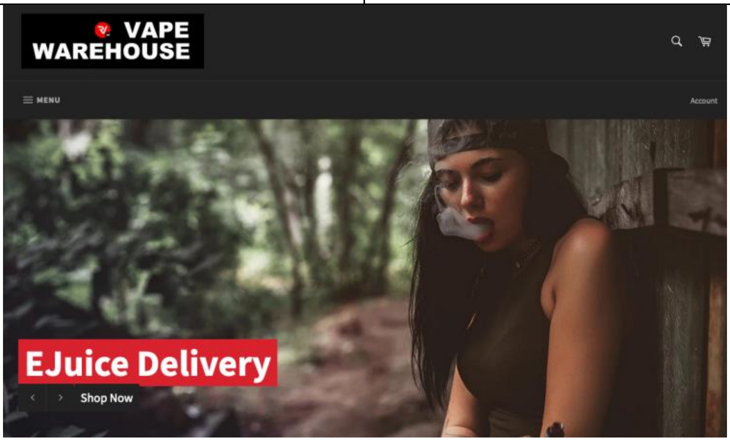
VAPE WAREHOUSE
MENU
Account
EJuice Delivery
Shop Now

Screenshots from websites catering to (left to right and top to bottom, starting from top left) Malaysia, Indonesia, Vietnam, Hong Kong, Taiwan and the Philippines. Bottom: front page of an online specialty e-cigarette store in the Philippines.
